# Aloin induced apoptosis by enhancing autophagic flux through the PI3K/AKT axis in osteosarcoma

**DOI:** 10.1186/s13020-021-00520-4

**Published:** 2021-11-24

**Authors:** Jiaming He, Wenkan Zhang, Xiaozhong Zhou, Weiqi Yan, Zhan Wang

**Affiliations:** 1 Department of Orthopedic Surgery, The Second Affiliated Hospital, Zhejiang University School of Medicine, Hangzhou, China; 2 The Second Affiliated Hospital, Zhejiang University School of Medicine, Hangzhou, China; 3 Key Laboratory of Motor System Disease Research and Precision Therapy of Zhejiang Province, Hangzhou, China

**Keywords:** Aloin, Network pharmacology, Molecular docking, Autophagy, Osteosarcoma

## Abstract

**Background:**

Osteosarcoma is a malignant tumor of bone and soft tissue in adolescents. Due to its tumor biological behavior pattern, osteosarcoma usually generates poor prognosis. Autophagy is an important self-defense mechanism in osteosarcoma.

**Methods:**

Cell viability in IC_50_ testing and reverse assays was examined by the MTT assay. Cell apoptosis conditions were examined by flow cytometry, Hoechst 33,342 staining and apoptosis-related protein immunoblotting. Autophagy conditions were tested by autophagy-related protein immunoblotting, transmission electron microscopic observation and dual fluorescence autophagy flux detection. The possible targets of aloin were screened out by network pharmacology and bioinformatic methods. Osteosarcoma xenografts in nude BALB/c mice were the model for in vivo research on tumor suppression, autophagy induction, pathway signaling and toxicity tests. In vivo bioluminescence imaging systems*,* immunohistochemical assays, and gross tumor volume comparisons were applied as the main research methods in vivo.

**Results:**

Aloin induced osteosarcoma apoptosis in a dose-dependent manner. Its possible effects on the PI3K/AKT pathway were screened out by network pharmacology methods. Aloin increased autophagic flux in osteosarcoma by downregulating the PI3K/AKT pathway. Aloin promoted autophagic flux in the osteosarcoma cell lines HOS and MG63 in a dose-dependent manner by promoting autophagosome formation. Chloroquine reversed the apoptosis-promoting and autophagy-enhancing effects of aloin. Autophagy induced by starvation and rapamycin significantly enhanced the autophagic flux and apoptosis induced by aloin, which verified the role of the PI3K/AKT axis in the pharmacological action of aloin. Therapeutic effects, autophagy enhancement and regulatory effects on the PI3K/AKT/mTOR pathway were demonstrated in a nude mouse xenogeneic osteosarcoma transplantation model.

**Conclusions:**

Aloin inhibited the proliferation of osteosarcoma by inhibiting the PI3K/AKT/mTOR pathway, increasing autophagic flux and promoting the apoptosis of osteosarcoma cells.

**Supplementary Information:**

The online version contains supplementary material available at 10.1186/s13020-021-00520-4.

## Introduction

Most osteosarcomas originate from primitive mesenchymal cells, which are derived from skeletal tissues [1, 2]. Osteosarcoma mainly harms adolescents and results in poor prognosis, which causes great social harm, and there is also a second smaller peak incidence in the elderly population [3, 4].

The mechanism of autophagy is worth exploring, and various targeted drug studies have highlighted the potential value of autophagy in clinical treatment [5–7]. Exploring important aspects of the autophagy mechanism in osteosarcoma may provide new ideas and insights for the treatment of osteosarcoma. In this study, we mainly focused on autophagy. On the one hand, autophagy is activated as a cytoprotective mechanism, providing nutrition and energy to tumor cells under starvation, thus increasing their resistance to common chemotherapy drugs. Autophagy also plays an important role in osteosarcoma suppression by inducing autophagic cell death [8, 9].

The occurrence of autophagy needs to be mediated by PI3KCIII, which is composed of the Vps34 complex [10, 11]. A series of cascade regulations are activated to promote mTOR phosphorylation to inhibit autophagy [10, 11]. mTOR is regulated by the PI3KC III-Vps34–Beclin-1-Vps15 complex to initiate autophagy [10–12]. When nutrients are rich, the PI3K/AKT signaling pathway activates mTOR after sensing glucose and amino acid concentrations to form the mTOR complex [11, 24], which degrades ULK1 and ATG13 through phosphorylation to hinder autophagosome formation [13–17]. When nutrients are deficient, ULK1 combines with ATG13 and RB1CC1 to transform the latter two into phosphorylated forms, competitively reducing ULK1 binding to mtorc1 and promoting autophagosome generation [13–17].

Aloin (National Center for Biotechnology Information (2021). PubChem Compound Summary for CID 12305761, Barbaloin. https://pubchem.ncbi.nlm.nih.gov/compound/Barbaloin.) is one of the main anthraquinone components extracted from aloe leaf secretion and is a natural C-glycoside of aloe anthrone. Aloin was reported to possess anti-inflammatory, antibacterial, antioxidant, antiviral and anticancer effects [18]. Studies have shown that aloin induces apoptosis in various tumor cells, including breast cancer [19], ovarian cancer [20, 21], B16-F10 mouse melanoma [22] and human Jurkat T lymphocytes [23].

Recent studies have shown that aloin has antitumor effects in various tumors. Its antitumor effects are closely related to cell growth inhibition, cell cycle arrest, and oxidative metabolic regulation.

Aloin induces tumor cell apoptosis through cell cycle arrest. Esmat applied aloin to breast and ovarian tumor cells to arrest the cell cycle in M phase and induce tumor cell apoptosis [19, 20]. Aloin reduced the mitotic ratio of cells by inhibiting the expression of topo IIα protein and downregulating cyclin B1 expression, thereby inducing the apoptosis of MCF-7 breast cancer cells [19, 20].

Nićiforović showed that aloin blocked the cell cycle of HeLaS3 cells in S phase and significantly increased the apoptosis rate [21]. Aloin interacted with CuZnSOD to produce an inhibitory effect, resulting in the accumulation of hydrogen peroxide to produce antiproliferative and cellular toxic effects [21].

Buenz found that after aloin treatment, Jurkat cells decreased in volume, impaired membrane integrity, and lost mitochondrial membrane potential [23]. Aloin induced melanoma cell differentiation by promoting melanin synthesis and transglutaminase activity in B16-F10 melanoma cells [22]. Considering the antitumor potential of aloin, we tried to study the chemotherapy effect of aloin in osteosarcoma, which is a relatively ‘tough’ tumor.

However, there is no literature report on the effect of aloin on human osteosarcoma and its underlying mechanism. Therefore, we used network pharmacology to study possible drug targets of aloin, which verified the antitumor effect on osteosarcoma in vitro and in vivo. In this study, we mainly discussed the autophagy induced by aloin in osteosarcoma cells and related PI3K/AKT/mTOR axis changes.

## Materials and methods

### Tumor cell lines

The selected cell lines were human osteosarcoma cell line MNNG/HOS (CRL-1547™, ATCC) and MG-63 (CRL-1427™, ATCC). All cell lines were cultured in Dulbecco’s modified Eagle’s medium (DMEM) with high glucose and L-glutamine (Corning), 10% fetal bovine serum (Gibco), 100 U/mL penicillin, and 100 μg/mL streptomycin (Sigma-Aldrich). The cells were incubated at 37 °C in a moist environment with 5% CO_2_, and then harvested and transferred to culture when they reached 70% to 80% confluence.

### Cell treatment

PBS, saline, and DMSO (Sigma-Aldrich) were used as vehicle and negative control for all treatments. Aloin (CAS:1415–73-2, 98.32% purity), Chloroquine (99.5% purity), Rapamycin (99.94% purity) (MedChemExpress) was applied for autophagic detection.

### MTT assay

Aloin cytotoxicity against HOS and MG-63 cell-lines was analyzed by MTT assay. HOS and MG-63 cell lines were treated with various concentrations of aloin for 24, 48, and 72 h. Triplicate experiments were performed and means ± SD was represented. The IC50s values of aloin in the HOS and MG-63 cells were calculated by Prism6.

### Hoechst 33342 staining detection

The two cell lines were cultured with different dose of aloin for 24 h, stained and photographed with fluorescence microscope under 488 nm. Cell apoptotic morphology was observed and photographed.

### Flow cytometry assays

After 24 h of aloin administration, HOS and MG63 cells were stained with Annexin V-FITC/PI Cell Apoptosis detection kit. FAC Scan flow cytometer (Beckman, USA) was used to analyze the samples and measure the percentage of apoptotic cells.

### Western blot assays

After 24 h of aloin, chloroquine, rapamycin and starvation administration, HOS and MG-63 cell lines were lysed in RIPA buffer with protease and phosphatase inhibitor cocktail. Protein samples of same amount were separated by western blot. Blot intensity was analyzed by Image J (v1.51). The primary antibodies applied in this research were as follows: AKT, ATG5, Beclin1, Bcl-2, bcl-xl, cleaved caspase 3, cleaved caspase 7, cleaved caspase 9, cleaved PARP, mTOR, pho-mTOR (SER2448), pho-AKT (SER473), PI3Kα (p110), SQSTM1 (P62), LC3B. Beta-actin was used as internal reference. The secondary antibodies were as follows: goat anti-rabbit IgG–HRP (1:2000, Biosharp) and goat anti-mouse IgG–HRP (1:2000, Biosharp).

### Network pharmacology and bioinformatics analysis on Aloin

The ligand structural data of aloin was obtained from PubChem (https://pubchem.ncbi.nlm.nih.gov/). Aloin 3D structure model file was loaded into the PharmMapper [42–44] (http://www.lilab-ecust.cn/pharmmapper/) website for reverse screening. The selection standard was as follows: human target protein, duplicate genes merged, fitscore from high to low sorted. Screened results were imported into protein–protein interaction network (protein–protein interaction, PPI) online analysis database STRING (https://string-db.org/) to analyze aloin-related drug targets interaction.

The obtained PPI network results were imported into Cytoscape (v3.6.0) to construct key network of aloin-target interactions. The obtained key network targets were uploaded to KEGG (https://www.kegg.jp/) for the construction of signaling pathway network. The three-dimensional structure of the target proteins (AKT1, CASP3, SRC, EGFR, MAPK8, MAPK14) for Chimera (v1.13) and Vina (v1.12) flexible matching with aloin were downloaded from the PDB database (http://www.rcsb.org/). The binding scores and H bonds were calculated.

### Autophagy flux detection

Mycoplasma elimination agent (Plasmocin, InvivoGen), penicillin and streptomycin were administrated for more than two weeks for elimination of microbial contamination before lentiviral transfection. Then the obtained mcherry-EGFP-LC3-puro-HOS and mcherry-EGFP-LC3-puro-MG63 cell lines were treated with different concentrations of aloin, chloroquine, rapamycin and starvation for 24 h. The slides were mounted under fluorescence microscope to enumerate the yellow (autophagosome) and red (autolysosome) fluorescent spots.

### Turnover experiments

To validate that Aloin-induced autophagy was modulated by PI3K/AKT/mTOR axis, we co-incubated aloin-treated HOS and MG-63 cells with chloroquine (autophagy inhibitor), starvation (autophagy synergy) and rapamycin (mTOR inhibitor). Cytotoxicity was analyzed by MTT assay. Apoptotic cells cluster was analyzed by flow cytometry. The protein expression levels of autophagic markers and key proteins of PI3K/AKT/mTOR axis were detected by western blot.

### Xenograft orthotopic model

Four-week-old female Balb/c nude mice, which were provided by the Shanghai Laboratory Animal Center of the Chinese Academy of Sciences, were raised under specific pathogen-free conditions. About 5 × 10^6^ stably transfected HOS cells (HOS-luc) for imaging were injected into the right tibia platform under inhalation anesthesia. After 7 days, based on the luminescence intensity measured by in vivo bioluminescence imaging system (IVIS), the remaining mice were randomly divided into three groups (four mice per group). Starting from day 7, mice in the negative control (NC) group received an intraperitoneal injection of 200 μL saline every two days as placebo. Mice in the Aloin group were administered every two days with 40 mg/kg Aloin in saline (0.8 mg per mouse in 200 μl saline) with 1% DMSO as hydrotrope, starting one day after tumor inoculation. The cis-platinum group received an intraperitoneal injection of 4 mg/kg cis-platin (MCE) in saline (80 μg per mouse in 200 μl saline) every three days. The sham group received mock operation in the tibia platform and received 200 μL saline intraperitoneal injection every 2 days. The luminescence intensity was recorded twice a week. After four weeks of treatment, all mice were euthanized. Tumors, spleens, hearts, livers, lungs, and kidneys were excised and prepared for IHC or HE slide staining.

### In vivo bioluminescence assay

The tumor-bearing mice were anesthetized with isoflurane for about 10 min after intraperitoneal injection of 150 μL of luciferin (20 mg/mL). In vivo imaging was implemented using an IVIS 200 imaging system and analyzed with Living Image Software (Version 3.0.4, Xenogen, Hopkinton, MA, USA).

### Histology and immunohistochemistry

Tumor, cardiac, hepatic, splenic, pulmonary and renal tissue slides were HE stained. Other tumor tissue slides were prepared for IHC. Slides were incubated with primary antibodies (LC3B, mTOR, pho-mTOR, AKT, pho-AKT, PI3Kα). The staining intensity was scored by ImageJ (v1.51).

## Statistical analysis

The results were described as the means ± SD. The data from groups was analyzed by ANOVA test. Pictures were processed by ImageJ (v1.51). Data and figures were managed by Graphpad Prism (v6.0). All statistics were analyzed by IBM SPSS Statistics 20.0 software (IBM, Armonk, NY, USA). Statistical significance was identified as P < 0.05. ****,***, **, and * represent P < 0.0001, P < 0.001, P < 0.01, and P < 0.05, respectively.

## Results

### Aloin induced HOS and MG-63 cell apoptosis in vitro

**Fig. 1:**
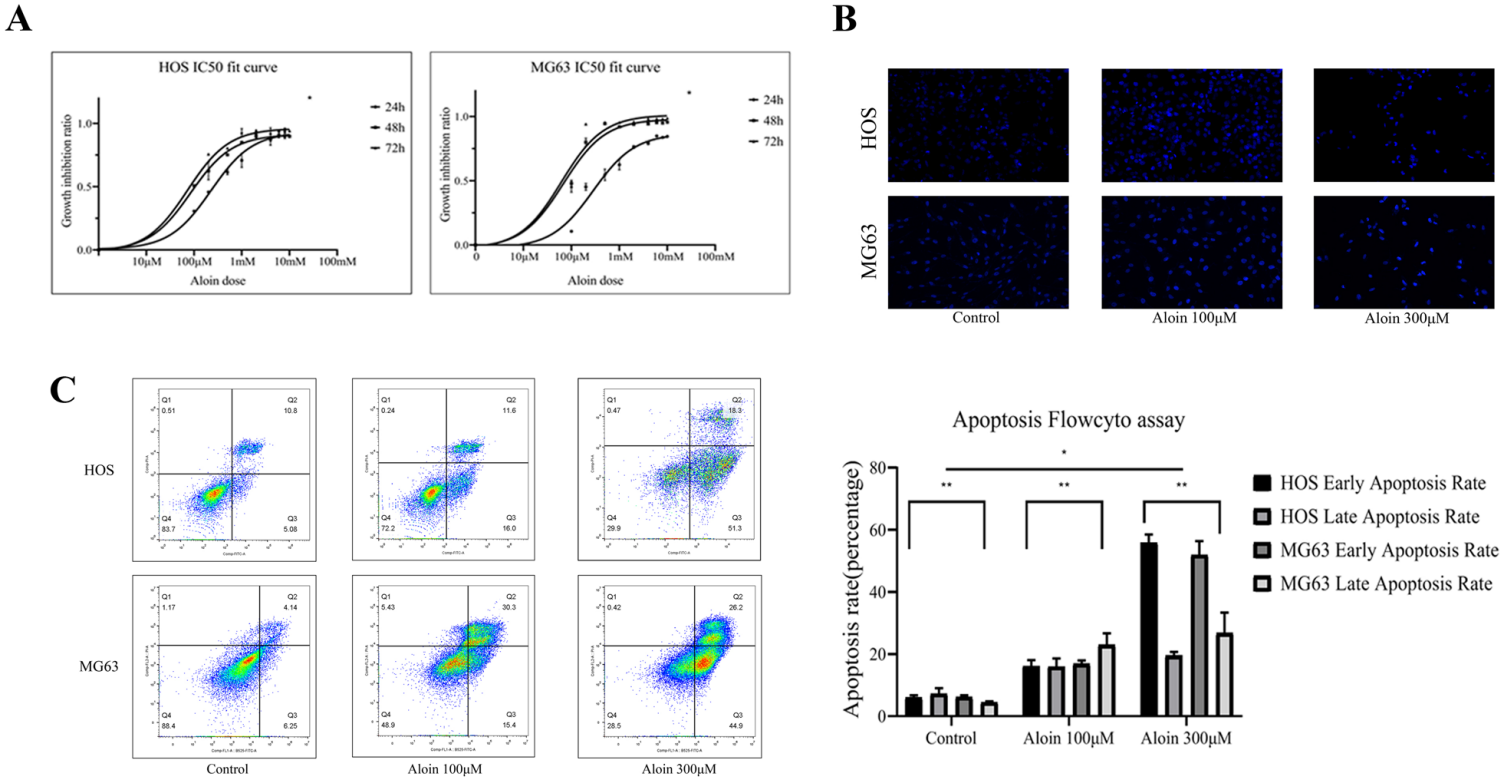
Aloin inhibited osteosarcoma cell proliferation in vitro. **A** Human osteosarcoma cell lines (HOS & MG63) were treated with different concentrations of aloin for 24, 48 or 72 h before MTT assays were performed. **B** HOS & MG63 cells were treated with different concentrations of aloin (0, 100 and 300 μM) for 24 h before Hoechst 33,342 staining was utilized for apoptosis morphology analysis. **C** HOS & MG63 cell lines were incubated with aloin for 24 h before the FITC-annexin V/PI apoptosis detection kit was utilized for flow cytometry. Error bar = mean ± SD of at least triplicate experiments. (* P < 0.005, **P < 0.01, ***P < 0.001, ****P < 0.0001)

Cell apoptosis was induced in both the human osteosarcoma cell lines HOS and MG-63 by aloin administration, which showed dose-and time-dependence (Fig. 1). The MTT cell viability assay was conducted to evaluate the inhibition rate curve of the cell lines. The IC_50_ values of aloin in the HOS cell line were 225.7 μM (24 h), 84.94 μM (48 h) and 72.89 μM (72 h). The IC_50_ values of aloin in the MG-63 cell line were 283.6 μM (24 h), 67.99 μM (48 h) and 62.28 μM (72 h) (Fig. 1A). The inhibition rate curve showed inhibition even at low doses, which was different from the feature of low-dose promotion in certain native compounds. With increasing doses, cell viability was significantly inhibited. Therefore, we chose 100 μM as the low-dose group and 300 μM as the high-dose group for further research.

The average apoptosis rate of the aloin groups (HOS:control 13.24%, low-dose 32.1%, high-dose 75.3%; MG-63:control 10.7%, low-dose 39.9%, high-dose 78.7%) was significantly higher than that of the control group (Fig. 1C). The early and late apoptosis clustering proportions showed obvious differences between HOS and MG-63 cells (Fig. 1C). Although the early apoptosis rate showed no large difference between the cell lines, a significant difference in late apoptosis existed between HOS and MG-63 cells. The apoptosis morphology was studied by Hoechst 33342 staining of slides under fluorescence microscopy observation. The increase in the concentration of aloin reduced the total cell number, and the proportion of densely stained cells increased significantly (Fig. 1B).

**Fig. 2:**
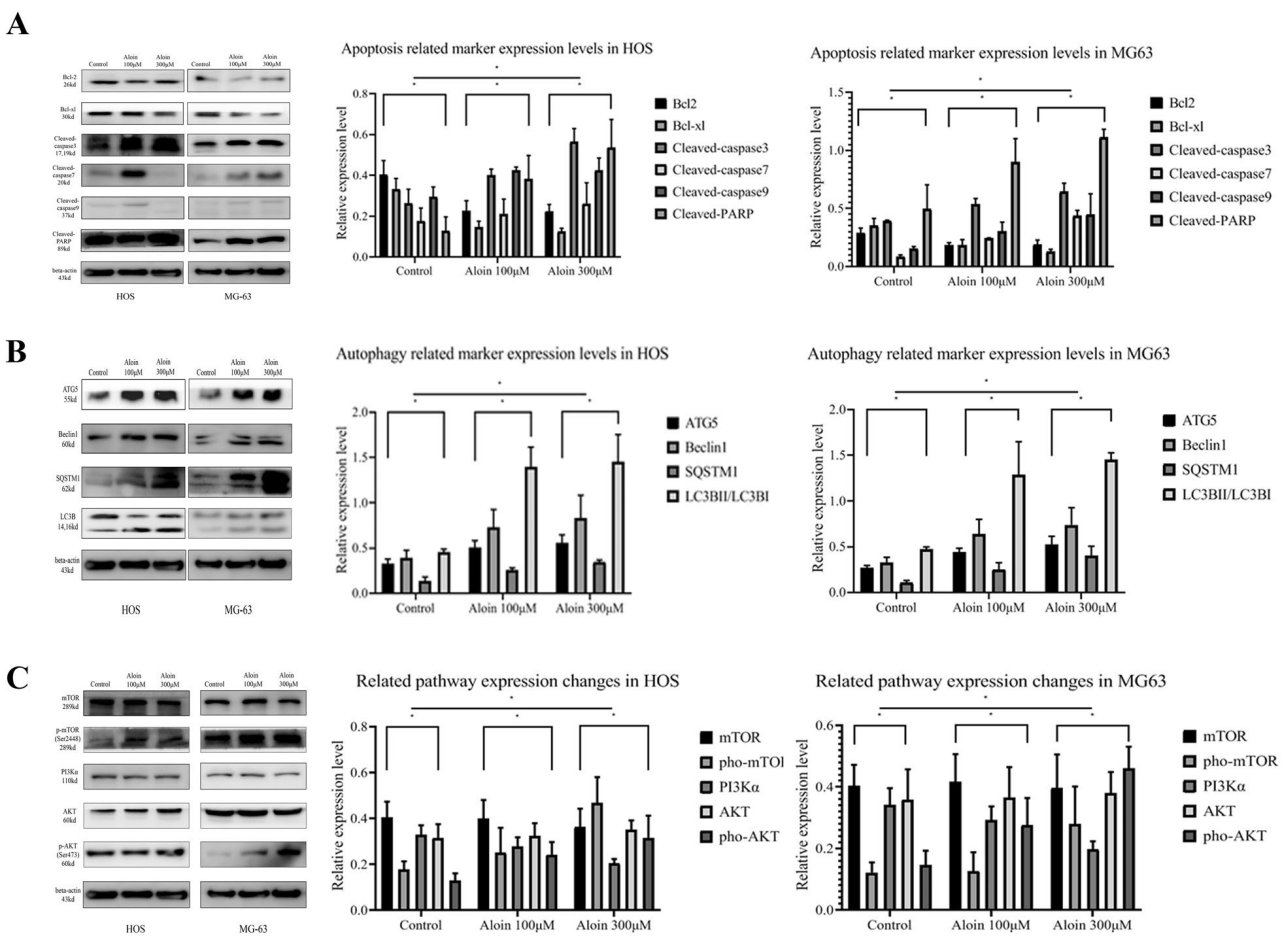
Aloin induced protein expression level changes in HOS and MG63 cell lines in vitro. The expression levels of apoptosis-related markers (**A**), autophagy-related markers (**B**) and PI3K/AKT/mTOR axis proteins (**C**) were determined by western blot after aloin administration (0, 100, and 300 μM) for 24 h. Western blot quantitative analysis was performed using ImageJ (ver 1.8). Error bar = mean ± SD of at least triplicate experiments. (*P < 0.005, **P < 0.01, ***P < 0.001, ****P < 0.0001)

Western blotting was used to analyze the expression of apoptosis-related proteins: cleaved PARP, cleaved caspase-3, cleaved caspase-7, cleaved caspase-9, Bcl-xl, and Bcl-2. The increasing expression levels of cleaved PARP, cleaved caspase-3, cleaved caspase-7, and cleaved caspase-9 indicated the initiation of the cell death process (Fig. 2A). The decreased Bcl-2 and Bcl-xL in the BCL family are antiapoptotic proteins (Fig. 2A). The apoptotic protease family is the junction of autophagy and cell death, which may play an important role in cell apoptosis through different pathways. We have reason to infer that the mechanism by which aloin promotes the apoptosis of osteosarcoma cells may involve the cross link between programmed death of tumor cells and autophagy.

### Network pharmacology and bioinformatics analysis on aloin

**Fig. 3:**
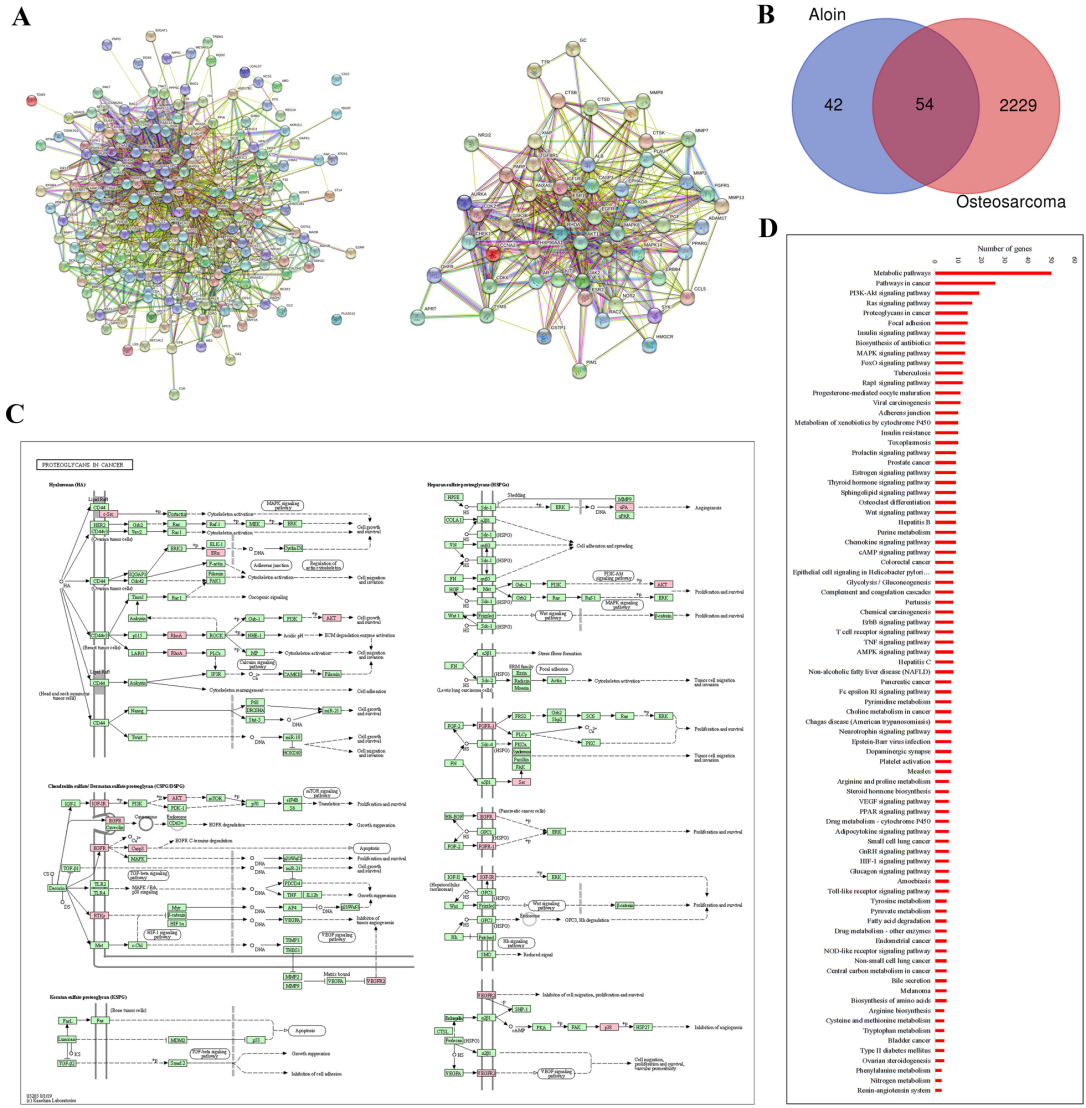
Network pharmacology and bioinformatics analysis on aloin. **A** Aloin-targeted proteins screened by PharmMapper were imported into STRING to analyze the interaction network and to construct a key network of aloin drug-target interactions. **B** The aloin target group and the osteosarcoma-related gene group were annotated to find the intersection of the groups. **C** Related pathways were screened from a key network of aloin by KEGG pathway analysis. **D** Aloin-related diseases, signaling pathways and biological processes were screened by the KEGG database. Visualization was performed by Cytoscape (ver 3.6.0), KEGG pathway and STRING databases

The annotated candidate genes were ranked in descending order with Fitscore scores by PharmMapper, and 196 candidates were selected for the next step of network construction. A total of 196 annotated aloin targets were imported into the STRING database to construct a protein interaction network (Additional file 1: Datasets). The STRING network analysis showed that 192 nodes interact with each other, 1317 edges can be obtained in the network, and 4 isolated nodes indicate that the target is not involved in interaction (Fig. 3A). In the PPI network structure diagram, AKT and EGFR in the core area had densely interacting lines.

The average degree was 13.7, and the average betweenness was 0.0076. There were 96 target genes whose midpoint degree centrality exceeded the median (median 9.5) in 196 nodes (Table 1). Among them, AKT, EGFR, CASP3, HSP90AA1, MAPK8, ESR1, MAPK14, JAK2, and PARP1 were found, suggesting that these proteins may be important targets for aloin to exert its pharmacological effects.

**Table 1:** Selcect Aloin targets and network topology parameter

Gene	Degree	Betweenness	Closeness
ALB	94	0.22467212	0.64726027
AKT1	82	0.10659732	0.61967213
EGFR	65	0.05548461	0.57446809
SRC	59	0.04756542	0.55752212
CASP3	57	0.04143446	0.5625
HSP90AA1	57	0.05023598	0.55588235
MAPK8	52	0.02459074	0.53846154
ESR1	52	0.03272838	0.53846154
RHOA	42	0.02871204	0.51780822
MAPK14	39	0.02442919	0.50670241
NOS3	38	0.02906078	0.52793296
ANXA5	36	0.00644395	0.50534759
AR	36	0.01980948	0.504
KDR	31	0.00735314	0.49606299
IGF1R	28	0.00777869	0.4871134
PGR	28	0.00682124	0.48586118
JAK2	26	0.00395944	0.48337596
XIAP	26	0.00384509	0.47848101
DHFR	26	0.03720463	0.48963731
SERPINA1	26	0.02045756	0.48461538
F2	25	0.0220183	0.47487437
NOS2	25	0.02107948	0.48963731
LCK	24	0.00374638	0.47848101
PRKACA	24	0.01605781	0.45215311
PARP1	24	0.00296672	0.47607053
HSPA8	24	0.00657807	0.48461538
SOD2	24	0.01434656	0.48461538
PTPN1	23	0.00625243	0.47848101
TYMS	23	0.02290566	0.46210269
CCL5	23	0.02444585	0.46782178
AKR1B1	23	0.01604294	0.4871134
REN	23	0.01096858	0.48091603
CTSD	23	0.01401781	0.4871134
CTSB	22	0.00521861	0.4713217
PPARG	22	0.01297883	0.47848101
GSK3B	22	0.0135452	0.47607053
MMP3	22	0.00435397	0.46898263
CDK2	22	0.00497381	0.47969543
CCNA2	21	0.00303664	0.47487437
ESR2	20	0.00923214	0.47368421
NQO1	20	0.01513652	0.48337596
LDHB	20	0.02011272	0.41906874
PLAU	20	0.01704925	0.46666667
APRT	20	0.01825685	0.41906874
HK1	20	0.01457167	0.47969543
CDK6	19	0.00306573	0.4375
GSR	19	0.00645307	0.48461538
GSTP1	19	0.018327	0.47368421
CHEK1	19	0.00214974	0.44575472
KIT	18	0.01193652	0.46323529
ADAM17	18	0.0033682	0.45985401
BACE1	17	0.00213241	0.45873786
MMP13	17	0.0012115	0.45873786
CASP7	17	7.17E−04	0.45985401
AURKA	17	0.00146614	0.4375
MMP7	17	8.03E−04	0.45873786
CTSG	16	0.00426012	0.43851508
MAPK10	16	0.01109269	0.45762712
TTR	15	0.00498248	0.43648961
CTSK	15	0.00240011	0.42857143
FGFR1	15	0.00103615	0.45
PGF	15	0.00283624	0.45542169
MMP8	15	0.0060175	0.45985401
SELP	15	0.00529684	0.45432692
CTNNA1	15	0.00111691	0.41629956
GC	14	0.00248048	0.43052392
RXRA	14	0.00553557	0.44893112
TGFBR1	13	0.00140318	0.44893112
RAC2	13	0.00111334	0.42281879
PDPK1	13	4.16E−04	0.43150685
FKBP1A	13	0.00421252	0.43249428
CDA	13	0.00590545	0.37724551
PCK1	13	0.00718678	0.45
RHEB	12	0.00261494	0.43052392
PNP	12	0.00616886	0.375
SULT2A1	12	0.00444855	0.43953488
LYZ	12	0.00507557	0.42954545
AKR1C3	12	0.00267438	0.39130435
EPHA2	11	0.00216285	0.421875
PAH	11	0.00349199	0.44158879
AHCY	11	0.00463874	0.40997831
RAB11A	11	0.01089449	0.44893112
RAB5A	11	3.99E−04	0.44158879
PDE5A	11	0.00318536	0.44055944
TYMP	11	0.00444257	0.42567568
NR1I2	11	0.00561541	0.44680851
F7	11	0.0018532	0.42281879
SYK	11	0.00397399	0.43249428
ERBB4	11	1.90E−04	0.42
HMGCR	11	0.00974638	0.4478673
HSD17B1	10	0.00143199	0.37649402
OTC	10	0.01325372	0.421875
PPIA	10	0.00163724	0.45432692
PIM1	10	0.01355121	0.43448276
CSNK2A1	10	0.00106061	0.41814159
SORD	10	0.00381009	0.43851508

The word ‘Osteosarcoma’ was searched in the Disgenet database (www.disgenet.org), disease number C0029463, and the screening condition was ‘Summary of GDAs’, with 2283 pieces of gene information obtained. The aloin target group (96 targets) and the osteosarcoma-related gene group (2283 targets) were annotated with the UNIPROT gene coding to find the intersection of both groups. A Venn diagram was generated according to the intersection (Fig. 3B). Fifty-four core targets were screened to form the key network genes (Table 2).

**Table 2:** Aloin and osteosarcoma intersection gene list (54 genes)

Gene name	Uniprot ID	Protein name
NOS2	P35228	Nitric oxide synthase, inducible
FGFR1	P11362	Fibroblast growth factor receptor 1
GSTP1	P09211	Glutathione S-transferase P
AURKA	O14965	Aurora kinase A
DHFR	P00374	Dihydrofolate reductase
GC	P02774	Vitamin D-binding protein
CTSB	P07858	Cathepsin B
KIT	P10721	Mast/stem cell growth factor receptor Kit
JAK2	O60674	Tyrosine-protein kinase JAK2
XIAP	P98170	E3 ubiquitin-protein ligase XIAP
CTSK	P43235	Cathepsin K
ESR1	P03372	Estrogen receptor
TYMS	Q53Y97	Thymidylate synthase
CCNA2	P20248	Cyclin-A2
HMGCR	P04035	3-hydroxy-3-methylglutaryl-coenzymeA reductase
MMP3	P08254	Stromelysin-1
MMP8	P22894	Neutrophil collagenase
PGF	P49763	Placenta growth factor
EGFR	P00533	Epidermal growth factor receptor
MAPK8	P45983	Mitogen-activated protein kinase 8
SRC	P12931	Proto-oncogene tyrosine-protein kinase Src
CDK2	P24941	Cyclin-dependent kinase 2
CCL5	P13501	C–C motif chemokine 5
ADAM17	P78536	Disintegrin and metalloproteinase domain-containing protein 17
REN	P00797	Renin
ALB	P02768	Albumin
ESR2	Q92731	Estrogen receptor beta
APRT	P07741	Adenine phosphoribosyltransferase
ERBB4	Q15303	Receptor tyrosine-protein kinase erbB-4
PLAU	P00749	Urokinase-type plasminogen activator
MMP13	P45452	Collagenase 3
EPHA2	P29317	Ephrin type-A receptor 2
RAC2	P15153	Ras-related C3 botulinum toxin substrate 2
CHEK1	O14757	Serine/threonine-protein kinase Chk1
PPARG	P37231	Peroxisome proliferator-activated receptor gamma
RHOA	P61586	Transforming protein RhoA
PIM1	P11309	Serine/threonine-protein kinase pim-1
MAPK14	Q16539	Mitogen-activated protein kinase 14
CDK6	Q00534	Cyclin-dependent kinase 6
TGFBR1	P36897	TGF-beta receptor type-1
CTSD	P07339	Cathepsin D
ANXA5	P08758	Annexin A5
HSP90AA1	P07900	Heat shock protein HSP 90-alpha
CASP3	P42574	Caspase-3
PARP1	P09874	Poly [ADP-ribose] polymerase 1
KDR	P35968	Vascular endothelial growth factor receptor 2
TTR	P02766	Transthyretin
SYK	P43405	Tyrosine-protein kinase SYK
IGF1R	P08069	Insulin-like growth factor 1 receptor
AKT1	P31749	RAC-alpha serine/threonine-protein kinase
NR1I2	O75469	Nuclear receptor subfamily 1 group I member 2
AR	P10275	Androgen receptor
MMP7	P09237	Matrilysin
GSK3B	P49841	Glycogen synthase kinase-3 beta

**Fig. 4:**
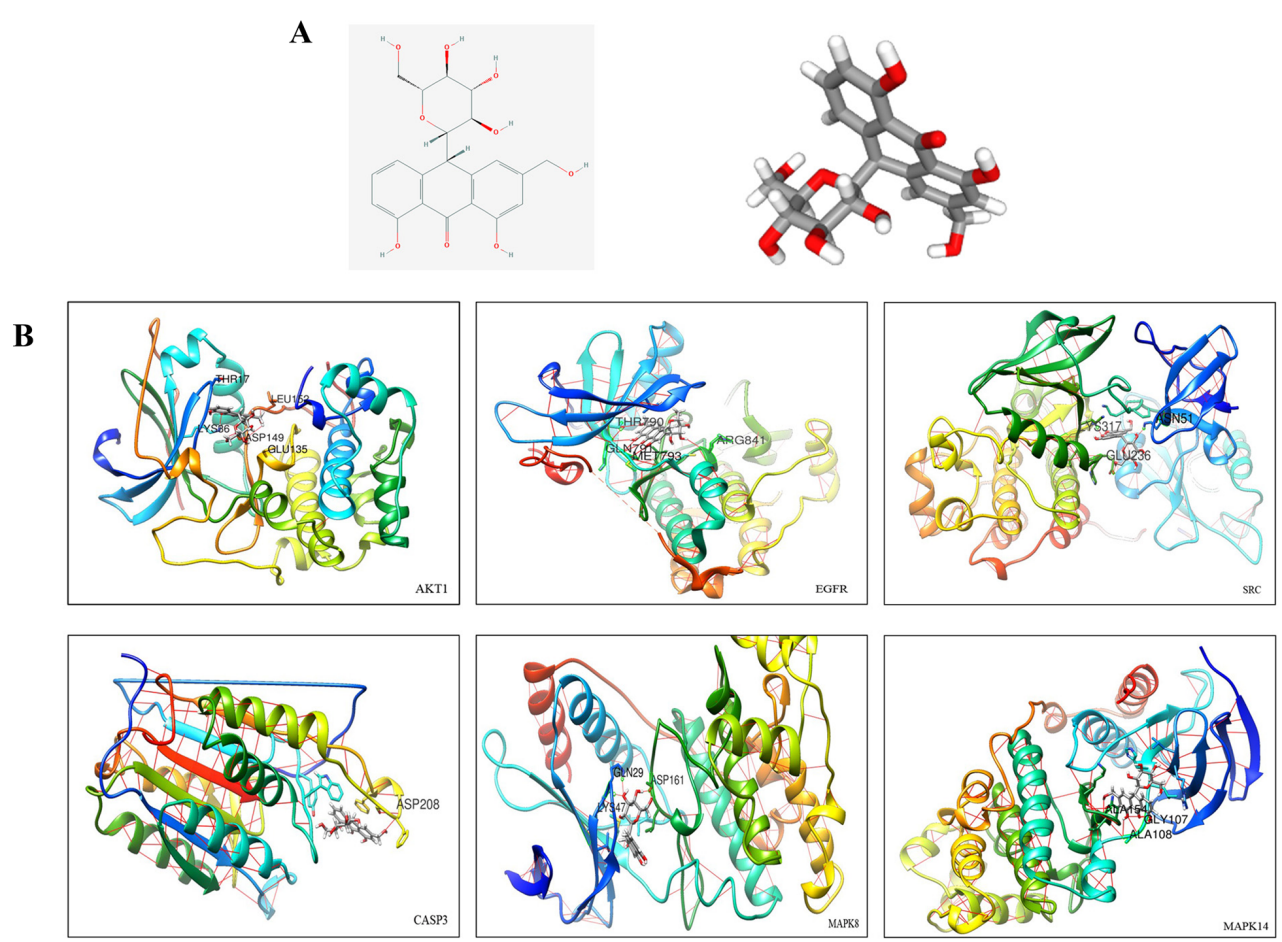
Docking of aloin. **A** 2D and 3D structure of aloin (From Pubchem). **B** Molecular docking of aloin with AKT1, EGFR, SRC, CASP3, MAPK8, and MAPK14. Docking and visualization by Chimera (v1.13) and Vina (v1.12)

A total of 59 closely related genes were screened in the signaling pathways enriched by KEGG, involving 127 related diseases, signaling pathways and biological processes (Fig. 3D). Among the 127 signaling pathways and biological processes, 24 signaling pathways were directly related to tumors, occupying a high proportion of 18.89% (24/127). There were 15 PI3K-AKT pathway-related targets and 14 MAPK pathway-related targets. Important nodes such as AKT1, CASP3, JAK2, MAPK8, MAPK14, and EGFR according to the degree of target association were mapped. Enrichment analysis of KEGG mapper and KEGG pathways found that multiple signaling pathways were related to the onset of cancers. AKT1, CASP3, EGFR, SRC, MAPK8, and MAPK14 were considered key proteins in the network (Fig. 3C). The 3D structures of the AKT1, CASP3, SRC, EGFR, MAPK8, and MAPK14 proteins were obtained from the PDB database (https://www1.rcsb.org/) and imported into Chimera (v1.13) with Vina (v1.12). The candidate targets were preprocessed and forward docked with the aloin molecule (Fig. 4B; Table 3). Molecular graphics and analyses were performed with UCSF Chimera, developed by the Resource for Biocomputing, Visualization, and Informatics at the University of California, San Francisco, with support from NIH P41-GM103311 [45].

**Table 3:** Aloin docking with key proteins

PDB ID	Score	Target gene	H bonds
3CQW	−8.0	AKT1	ASP149(2.132 Å,2.223 Å), LEU152(1.875 Å), LYS36(1.965 Å)
2ITX	−8.3	EGFR	GLN791(2.079 Å,2.628 Å), MET793(2.264 Å, 2.353 Å,2.333 Å), ARG841(2.242 Å), THR790(2.216 Å)
2H8H	−8.4	SRC	ASN51(1.930 Å), LYS317(2.191 Å), GLU236(2.010 Å)
3DEJ	−7.6	CASP3	ASP208(2.574 Å)
1UKI	−8.0	MAPK8	ASP161(1.854,1.526 Å), CYS47(1.930 Å), GLN29 (2.226 Å)
1W84	−8.0	MAPK14	GLY107(2.274 Å,2.108 Å), ALA108(2.204 Å, 2.274 Å), ALA154(2.304 Å)

Based on the abovementioned network pharmacology research results, the regulatory effect of aloin was likely to be closely related to key pathway proteins such as AKT1, EGFR and CASP3. PI3K-AKT-related pathways were also closely involved in the results. We considered the autophagy process, which is closely related to the PI3K/AKT/mTOR pathway, to be the keystone to study the antitumor mechanism of aloin.

### Aloin upregulated autophagic flux in HOS and MG-63 cells in vitro

Western blotting was conducted to detect autophagy protein markers, such as ATG5, P62, Beclin-1, and the ratio of LC3BI and LC3BII protein expression in the aloin-administered cultivation system. Aloin significantly increased the ratio of LC3BII/LC3BI in a dose-dependent manner (Fig. 2B). Before aloin treatment, the ratio of LC3BII/LC3BI in the control group was less than one, which indicated that the control group did not enter the initial process of autophagy. In the aloin groups, the expression of LC3BII and LC3BI increased compared with that in the control group, and the LC3BII/LC3BI ratio of the groups was found to be greater than one, indicating that after aloin treatment, LC3BI transformed into LC3BII and aloin could increase autophagy. At the same time, we measured the expression levels of the autophagy marker proteins ATG5 and Beclin-1. Both expressions increased with increasing aloin concentration. The above results fully demonstrated that aloin significantly promoted the autophagy production process (Fig. 2B). At the same time, P62/SQSTM1 expression was increased. Increased Sequestosome-1 usually represents autophagy inhibition [30]. In actual research processes, an increase in P62 is commonly observed when autophagy is activated, especially under drug stimulation and oxidative stress [25].

**Fig. 5:**
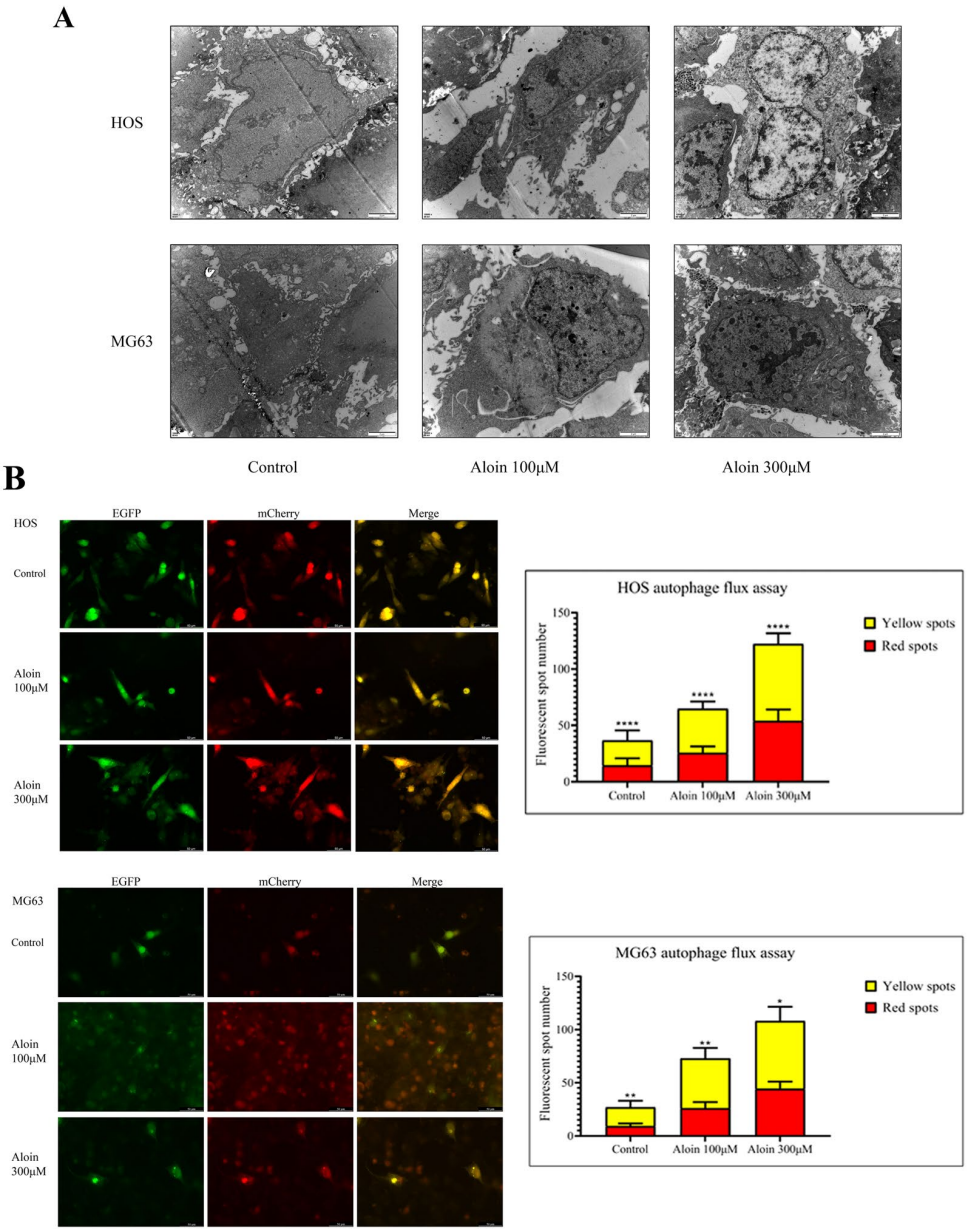
Autophagy assay in aloin-treated HOS & MG63 cells. **A** Transmission electron microscopy scanning of aloin-treated HOS & MG63 cells to observe autophagosome formation. **B** Dual-fluorescence autophagy flux assay in aloin-treated HOS & MG63 cells. The wavelength was 488 nm and 560 nm. A fluorescence spot count was applied by ImageJ (v1.8). Error bar = mean ± SD of at least triplicate experiments. (* P < 0.005, **P < 0.01, ***P < 0.001, ****P < 0.0001)

To clarify the specific effect of aloin on autophagy, a combination of multiple research methods was needed to evaluate autophagy flux changes. Autophagosome dynamic formation, autophagosome-lysosome binding, and autophagy substrate degradation were also detected (Fig. 5A).

After treating osteosarcoma cell lines with different concentrations of aloin, both the autophagosomes and autophagolysosomes counted under a fluorescence microscope increased. This phenomenon was in line with the increasing trend of the LC3B protein expression level measured in the western blot experiment. Although the expression of P62/SQSTM1 increased, increased degradation did not significantly inhibit the overall autophagic flux. Aloin increased the autophagic flux in a dose-dependent manner, which mainly relied on an increase in autophagy (Fig. 5B).

### Aloin induced autophagy-related apoptosis in HOS and MG-63 cells via the PI3K/AKT/mTOR axis

Aloin might increase autophagic flux through the PI3K/AKT/mTOR pathway in HOS and MG-63 cell lines. Our experiments show that aloin-related PI3K/AKT/mTOR axis regulation inhibited the growth of tumor cells and induced autophagy. In the previous part of the study, network pharmacology research and gene enrichment studies found that active aloin pharmaceutical targets were closely related to the PI3K/AKT signaling pathway. Therefore, we inferred that aloin may regulate osteosarcoma through this pathway. Western blot analysis was performed to determine the expression levels of mTOR, phosphorylated mTOR, regulatory subunit PI3Kα, AKT, and phosphorylated AKT. Aloin downregulated the expression of PI3Kα in a dose-dependent manner and upregulated the phosphorylation levels of mTOR and AKT at 100 and 300 μM (Fig. 2C).

On this basis, we applied methods to induce autophagy in osteosarcoma cells, inhibit the autophagy process of osteosarcoma cells, and inhibit the phosphorylation level of mTOR by rapamycin to clarify the relationships among aloin, autophagy, the PI3K/AKT/mTOR axis and apoptosis. Apoptosis, autophagy markers and PI3K/AKT/mTOR proteins were tested by western blot assay to observe the expression changes under different conditions. The MTT assay and flow cytometry were carried out to determine growth viability and apoptotic cell clusters of osteosarcoma in all regulatory groups. Dual fluorescence autophagy flux detection analysis of the autophagic flux in the three regulatory groups was performed.

**Fig. 6:**
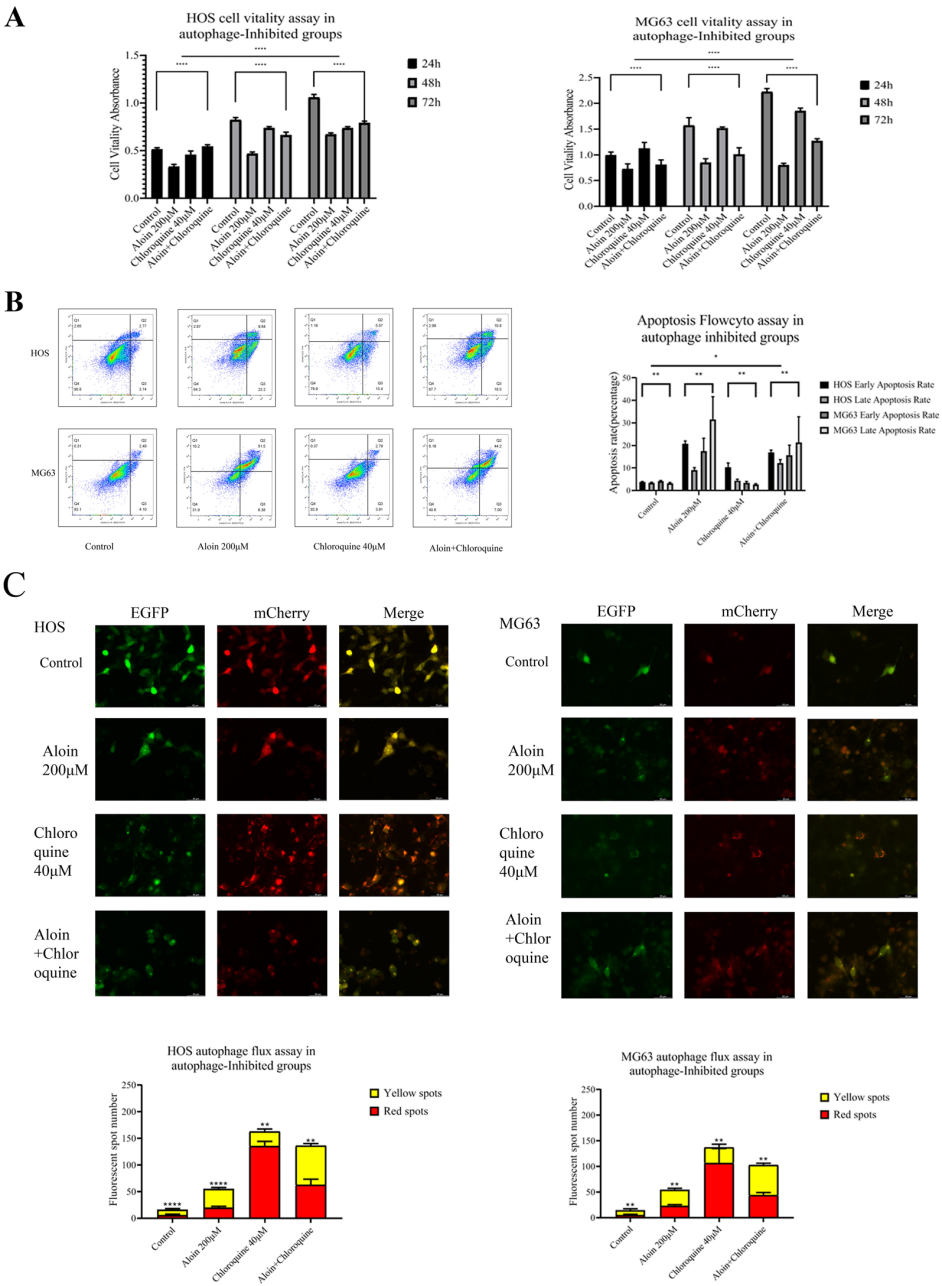
Aloin and chloroquine coadministration for turnover assays in HOS and MG63 cell lines. **A** After 24 h of treatment, MTT assays (**A**), flow cytometry assays (**B**), and dual fluorescence autophagy flux assays were carried out. Dual fluorescence spot quantitative analysis was performed by ImageJ (v1.8). Flowcyto clustering was applied by FLOWJO 10. Error bar = mean ± SD of at least triplicate experiments. (* P < 0.005, **P < 0.01, ***P < 0.001, ****P < 0.0001)

**Fig. 7:**
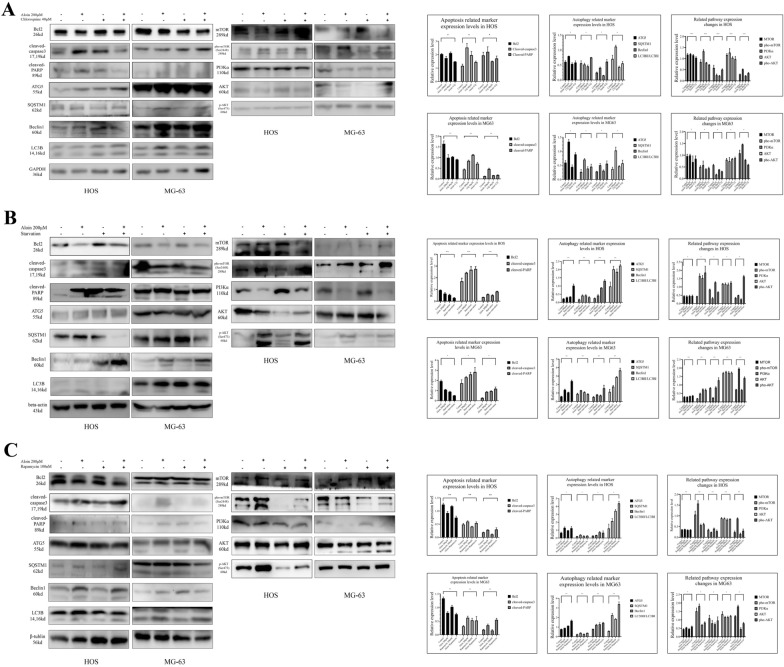
Protein expression levels in the aloin-chloroquine (**A**), aloin-starvation (**B**), and aloin-rapamycin (**C**) treatment groups. Apoptosis-related protein markers, autophagy-related proteins and PI3K/AKT/mTOR axis protein expression were tested by western blot. Western blot quantitative analysis was performed by ImageJ (v1.8). Error bar = mean ± SD of at least triplicate experiments. (* P < 0.005, **P < 0.01, ***P < 0.001, ****P < 0.0001)

**Fig. 8:**
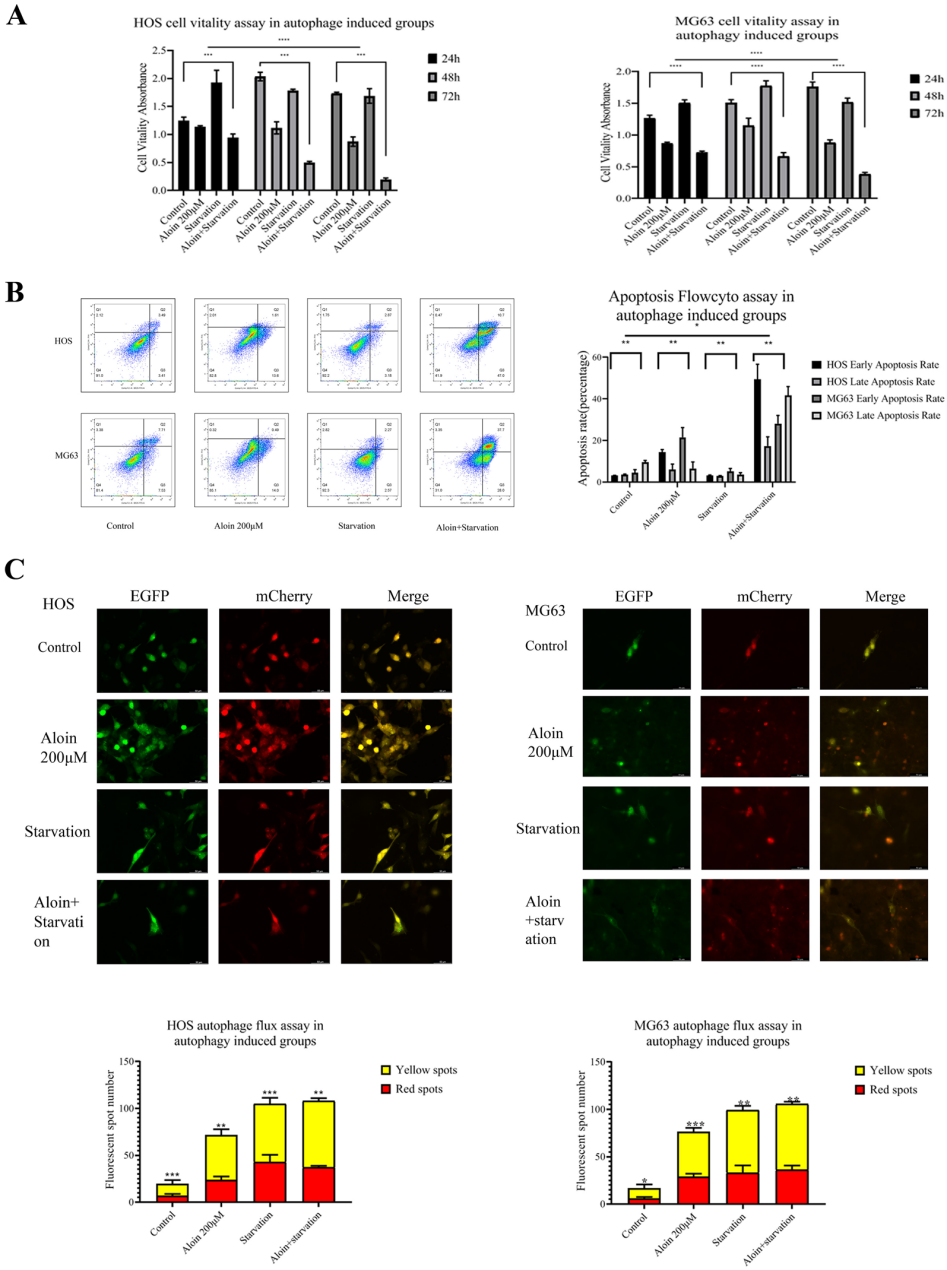
Aloin and rapamycin coadministration for the mTOR inhibition test in HOS and MG63 cell lines. After 24 h of treatment, MTT assays (**A**), flow cytometry assays (**B**), and dual fluorescence autophagy flux assays were carried out. Dual fluorescence spot quantitative analysis was performed by ImageJ (v1.8). Flowcyto clustering was applied by FLOWJO 10. Error bar = mean ± SD of at least triplicate experiments. (* P < 0.005, **P < 0.01, ***P < 0.001, ****P < 0.0001)

We found that when chloroquine and aloin were both administered to the culture system for 24 h, the growth activity of osteosarcoma cells recovered to a certain extent, and the proportions of apoptotic clusters showed a slight decline (Fig. 6A, B). Western blotting assays indicated that apoptosis and autophagy markers were decreased (Fig. 7A). The expression levels of downregulated PI3Kα recovered, and the upregulated phosphorylated levels of mTOR and AKT were reversed (Fig. 8A). Dual fluorescence autophagic flux detection found that chloroquine significantly inhibited the autophagic flux induced by aloin. Using a fluorescence microscope, it was observed that the number of yellow spots decreased, and the number of red spots increased, which reversed the yellow and red spots augmented induced by aloin alone (Fig. 6C).

To further investigate the biological effects of aloin on autophagy, we found that when the cells were starved, starvation had a synergistic antitumor effect. After the dual administration of aloin and starvation for 24 h, the proliferation ability of osteosarcoma cells was obviously inhibited, and the proportion of apoptotic cells obviously increased (Fig. 8A, B). Western blot analysis found that apoptosis marker expression was promoted and autophagy-related proteins were increased. Downregulated PI3Kα rose to a certain extent, and phosphorylated levels of mTOR and AKT were further upregulated (Fig. 7B). Dual fluorescence autophagic flux detection found that the synergistic effect of starvation and aloin led to a further augmentation in the autophagic flux. As observed with a fluorescence microscope, the number of yellow and red spots in the dual-administration group increased more significantly than in the starvation or aloin group alone (Fig. 8C).

**Fig. 9:**
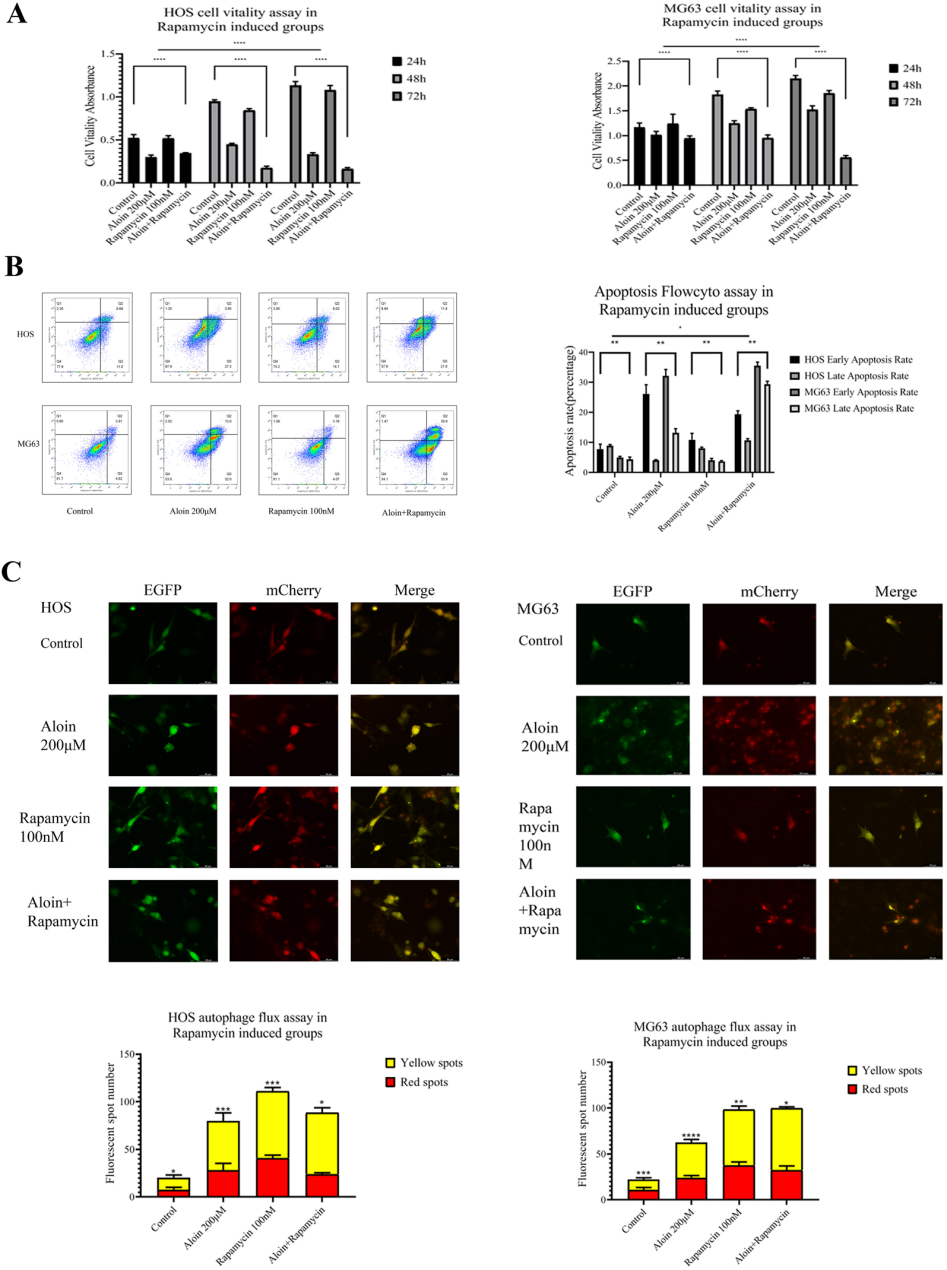
Aloin and starvation coadministration for the autophagy synergetic assay in HOS and MG63 cell lines. After 24 h of treatment, MTT assays (**A**), flow cytometry assays (**B**), and dual fluorescence autophagy flux assays were carried out. Dual fluorescence spot quantitative analysis was performed by ImageJ (v1.8). Flowcyto clustering was applied by FLOWJO 10. Error bar = mean ± SD of at least triplicate experiments. (* P < 0.005, **P < 0.01, ***P < 0.001, ****P < 0.0001)

Aloin significantly upregulated the phosphorylation levels of mTOR and AKT while promoting autophagy, inhibiting tumor cells, and downregulating PI3Kα. To further study the aloin-induced upregulation of phosphorylated mTOR and AKT, rapamycin was applied to inhibit mTOR. When rapamycin was applied as a mTOR inhibitor in this turnover experiment, phosphorylation levels of AKT and mTOR were downregulated by rapamycin, while the inhibition of PI3Kα was not relieved (Fig. 7C). Downregulated phosphorylation levels of AKT and mTOR did not inhibit autophagy. In the rapamycin administration group alone, the mTOR blockade induced by rapamycin returned to PI3Kα expression and promoted its expression. However, this effect did not reverse the PI3Kα inhibition induced by aloin in the dual-administration group. The final biological effect of the combined administration was still the inhibition of cell growth and autophagy induction (Fig. 9). The final inhibition effect was enhanced by the synergy of aloin and rapamycin (Figs. 9A, B).

### Aloin inhibits osteosarcoma in a mouse xenograft model of osteosarcoma

**Fig. 10:**
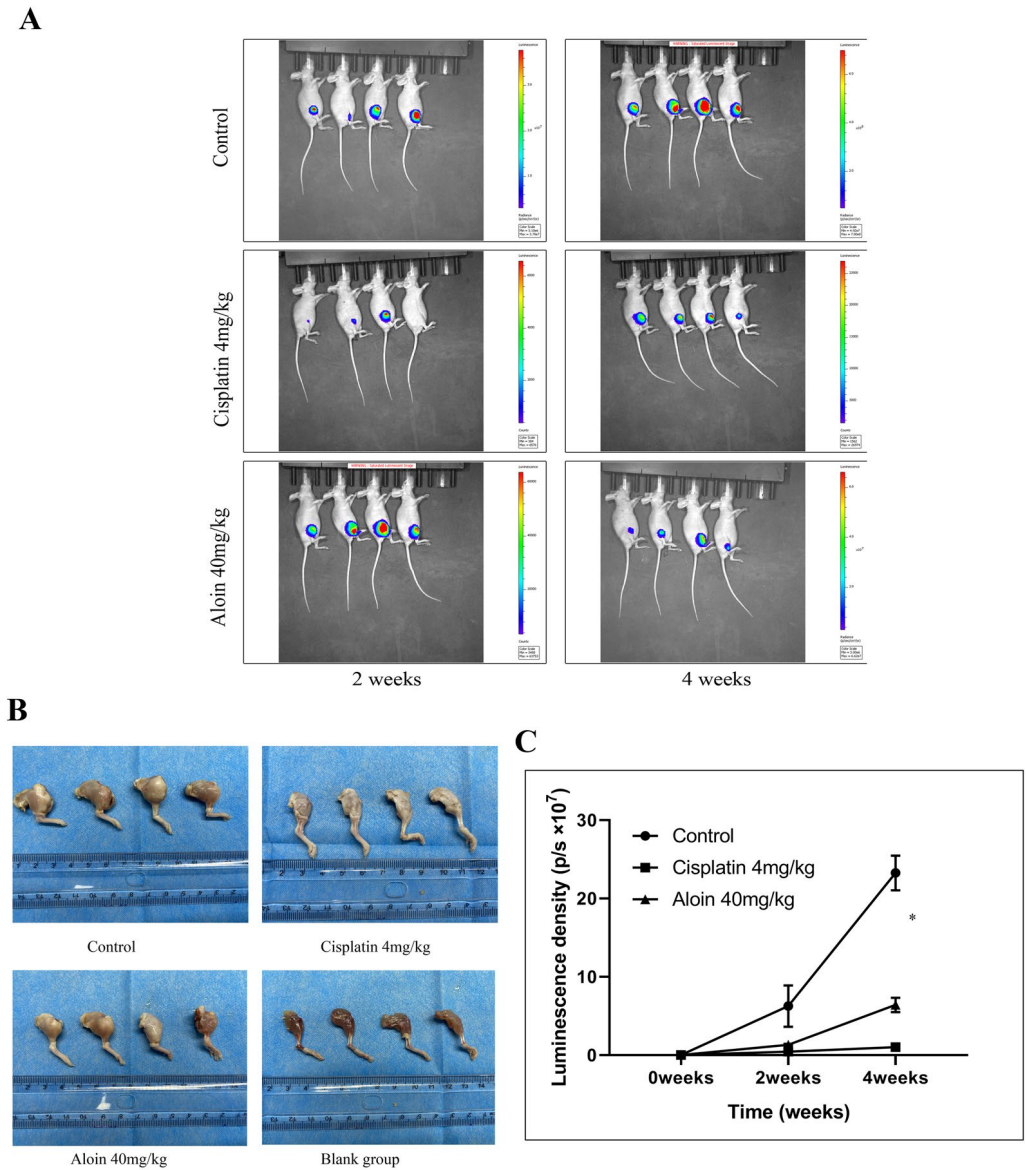
Aloin inhibited osteosarcoma growth in a xenograft model in BALB/c nude mice. **A** After 4 weeks of aloin treatment, the luciferase intensity of the tumors was measured at week 2 and week 4. **B** Gross observation showed significant differences among the control, aloin and cisplatin groups after 4 weeks. **C** IVIS intensity was measured by Living Image Software (v3.0.4). The cisplatin group was set for comparison, and the blank group was set as the sham-operated group. Error bar = mean ± SD of at least triplicate experiments. (* P < 0.005, **P < 0.01, ***P < 0.001, ****P < 0.0001)

In the Balb/c nude mouse xenograft model of osteosarcoma, aloin obviously inhibited human osteosarcoma cell (HOS) growth. Compared with the control group, the gross tumor volume in the aloin group was smaller. Compared with the cisplatin group, the gross tumor volume in the aloin group was slightly larger (Fig. 10B, C). The IVIS assay showed that the average fluorescence intensity of the control group was the highest. The luminescence intensity of the cisplatin group was significantly lower than that of the control group 7 days after administration. The difference in luminescence intensity was obvious at the end of the 4-week experiment. The luminous intensity of the aloin group was significantly lower than that of the control group after 7 days of administration, but the degree of reduction was less than that of the cisplatin group. At the end of the 4-week experiment, the luminescence intensity of the aloin group was significantly lower than that of the control group, but the overall decrease was still less than that of the cisplatin group. HE staining showed that the cisplatin group shared the highest proportion of necrotic tumor cells. The proportion of necrotic tumor cells in the aloin group was slightly lower than that in the cisplatin group but was significantly higher than that in the control group (Fig. 10A).

**Fig. 11:**
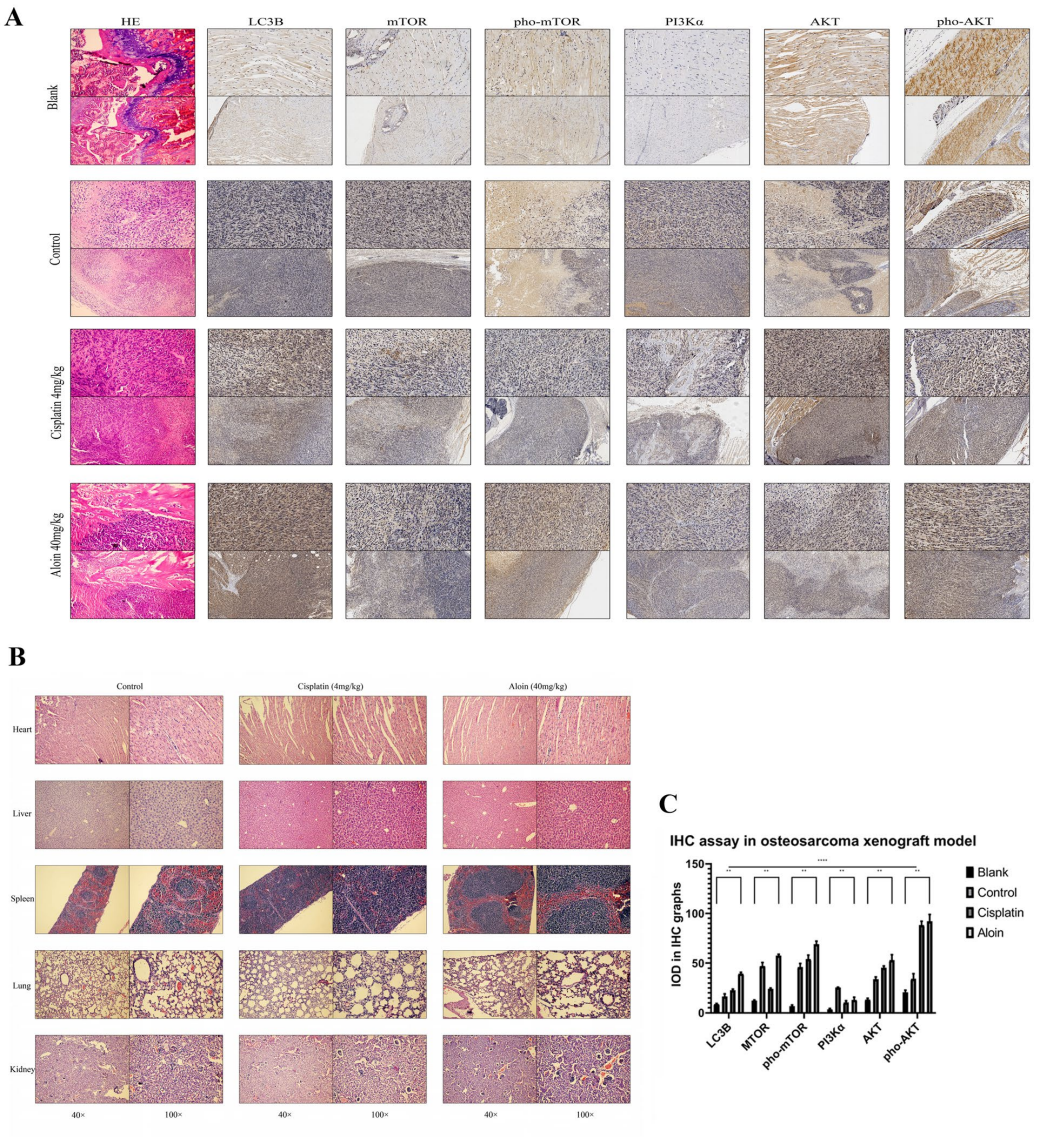
Histological detection in organs and tumors. **A** IHC and HE staining were utilized to assess tumor tissue sections. The markers examined were LC3B, mTOR, pho-mTOR, PI3Kα, AKT, and pho-AKT. **B** Toxic effects on organs were tested by HE staining. **C** The expression levels of each marker were quantified by ImageJ (v1.8). Magnification, × 200 (enlarged pictures), × 40 (unenlarged pictures). Error bar = mean ± SD of at least triplicate experiments. (* P < 0.005, **P < 0.01, ***P < 0.001, ****P < 0.0001)

Further immunohistochemical analysis confirmed that LC3B, phosphorylated AKT and phosphorylated mTOR expression induced by aloin was increased, while PI3Kα expression decreased to a certain extent (Fig. 11A and C). As a commonly used chemotherapeutic drug, cisplatin also causes a certain degree of autophagy in tumor cells. Therefore, LC3B expression in the cisplatin group also increased. Phosphorylated AKT and phosphorylated mTOR expression in the cisplatin group rose to a certain level. However, the increase in the LC3B amplitude in the cisplatin group was lower than that in the aloin group (Fig. 11A and C). These results indicate that aloin inhibited the growth of tumor cells in osteosarcoma xenograft tumor models and induced autophagy in vivo. Its regulatory effect on the PI3K/AKT/mTOR pathway was also in line with the test results in the in vitro cell culture system.

### Aloin toxicity test in vivo

H&E staining showed that aloin had no obvious toxic effects on the mouse heart, liver, spleen, lungs, or kidneys (Fig. 11B).

## Discussion

Most osteosarcomas originate from primitive mesenchymal cells, which are spindle cells that can differentiate to produce bone tissue. Most of these cells are derived from skeletal tissue, which may transform into malignant tumors [1, 2]. Osteosarcoma has a higher incidence during adolescence, which results in the annual incidence rate of its adolescent population as high as 8 to 11 cases/million/year, accounting for 8.9% of childhood cancer-related deaths [3]. In addition, a second smaller peak of incidence was found in the elderly [4].

The treatment of primary tumors in osteosarcoma is mainly chemotherapy and surgery [26]. After multidrug chemotherapy starts, the long-term survival rate increases to 70% [27, 28]. In addition to the therapeutic effect of chemotherapeutics, there were still some problems, such as toxicity to normal tissues, drug resistance and rapid clearance of blood drugs [29].

Aloin inhibited osteosarcoma cell proliferation by inducing apoptosis in a dose- and time-dependent manner. Therefore, it was necessary to clarify the mechanism by which aloin inhibited osteosarcoma growth from the perspective of pharmacological properties. We found that as the aloin concentration increased, the total cell number decreased, while the proportion of densely stained cells increased significantly. In this study, the expression levels of activated caspase-3, caspase-7, caspase-9, cleaved PARP, Bcl-2 and Bcl-xl were detected by Western blotting to study the mechanism by which aloin promotes osteosarcoma apoptosis. With increased aloin concentrations, the expression of cleaved-PARP and caspase-3, caspase-7, and caspase-9 increased, which induced apoptosis.

Members of the caspase family of proteins interact with autophagy-related proteins [31]. For example, caspase-3 inhibited autophagy by cleaving Beclin-1 to inactivate it. Increased Beclin-1 expression in turn promoted caspase-9 activity and promoted the occurrence of apoptosis [31, 32]. The mechanism by which aloin promotes apoptosis of osteosarcoma cells may involve the cross link between programmed death of tumor cells and autophagy.

We divided the antitumor target screening strategy of aloin into six steps. 1. Reverse virtual screening of potential targets for drug molecules through the PharmMapper database; 2. Protein interaction analysis; 3. KEGG pathway enrichment cluster analysis and key network construction: enrichment analysis screens and optimizes important sites involved in autophagy regulation and antitumor-related mechanisms; 4. Forward molecular docking research verification: forward molecular docking analysis potential target binding score; 5. Verification in in vitro cell culture system; and 6. Validation in animal models. Pharmacophore matching reverse screening of the possible targets of the antitumor effect of aloin was regarded as the starting point for preliminary screening research.

To reduce the probability of false-positive targets, previous studies were based on the Fitscore or Normal Fitscore, according to the purpose and means of the research [40, 41]. Combined with known sites of previous studies, we selected the PI3K/AKT/mTOR axis as a key research checkpoint and verified its pathway changes through experiments such as western blotting.

It was more effective to study interactions between small molecule compounds and signal pathway networks through bioinformatics methods [33]. This research strategy has become a common method for the efficient manufacturing of various inhibitors and activators [34]. This research evidence gave us reason to believe that these protein targets might be the key targets of aloin. Therefore, we extracted AKT, EGFR, CASP3, SRC, MAPK8, and MAPK14 for the next step of molecular docking verification.

Among the 6 target proteins, AKT1 had a considerable match score (-8.2), indicating that aloin was a firm and stable ligand that binds to AKT1. Compared with targets other than AKT1, SRC and EGFR also showed higher matching levels of -8.4 and -8.3, respectively, while MAPK8 and MAPK14 scored -8.0, and CASP3 scored -7.6. The PI3K/AKT signaling pathway, which we finally focused on, had important regulatory functions in oxidative stress, autophagy, metabolism, growth, proliferation, survival, transcription and protein synthesis. As important cancer regulatory nodes, EGFR and SRC also cross-talk with the PI3K/AKT pathway [35]. JNK (MAPK8), an important part of the MAPK signaling pathway, participates in processes such as apoptosis, oxidative stress, autophagy, and inflammation [36]. The PI3K/AKT axis was closely related to the occurrence of autophagy. Therefore, we decided to discuss autophagy as the key node in this study.

Aloin treatment of osteosarcoma cells increased the autophagic flux in a dose-dependent manner. We administered chloroquine (autophagy inhibitor), rapamycin (mTOR inhibitor), and starvation treatment to the culture system for further studies. Chloroquine inhibited aloin-induced apoptosis to a certain degree, while starvation and rapamycin treatment synergistically enhanced aloin’s induction of autophagy and apoptosis.

In this study, the formation of autophagosomes and the expression of autophagy markers after aloin treatment of osteosarcoma cells were detected. Upregulation of Beclin-1 and changes in the ratio of LC3I and LC3II were considered signs of increased autophagic flux [37, 38]. We found that aloin upregulated Beclin-1, increased LC3I and LC3II expression and promoted the conversion from LC3I to LC3II. Chloroquine reduced aloin-induced apoptosis to a certain extent by inhibiting autophagy. Therefore, the chloroquine turnover test proved that aloin promoted apoptosis of osteosarcoma cells by inducing autophagy.

After chloroquine and aloin were used to cotreat HOS and MG63 cells, apoptosis-related proteins were downregulated in the cotreatment group compared with the separate treatment groups and control group. The trends of autophagy-related proteins ATG5, P62, LC3B, Beclin-1, etc. also reflected that chloroquine inhibited the autophagy enhancement effect of aloin. ATG5 and LC3B exhibited more obvious inhibitory effects, while P62 and Beclin-1 showed slightly lower suppressive effects. After cotreatment with aloin and chloroquine, autophagolysosomes were reduced compared with those of the chloroquine group, while autophagosomes increased significantly. This trend was also consistent with the results of changes in autophagy-related protein expression.

Aloin treatment promoted the phosphorylation of AKT and mTOR, indicating the activation of AKT and mTOR. AKT and mTOR are two classic cancer-promoting pathways and are regarded as downstream sites of PI3K/AKT/mTOR [39]. The activation of mTOR inhibited autophagy, while mTOR inhibition often enhanced the process of autophagy [39]. Therefore, in follow-up studies, we conducted further studies on the effects of aloin, chloroquine, starvation treatment and rapamycin on the PI3K-AKT-mTOR signaling pathway in osteosarcoma cells.

Starvation treatment was applied to synergize with aloin-promoted autophagy, and rapamycin was used as a mTOR inhibitor to reverse aloin-promoted mTOR phosphorylation. Autophagosomes in the aloin starvation treatment groups were significantly increased. Cell viability was significantly inhibited, and the apoptosis ratio was significantly increased. Western blot detection also showed that the expression of apoptosis-related markers and autophagy-related proteins increased significantly. The PI3K/AKT/mTOR axis was further inhibited to a certain extent, in which PI3Kα was inhibited.

When rapamycin was used as a mTOR inhibitor, aloin-promoted AKT and mTOR phosphorylation was inhibited by rapamycin, while PI3Kα inhibition induced by aloin was not relieved. In the rapamycin alone group, rapamycin-blocked mTOR returned to the expression of PI3Kα to promote its upregulation. However, this effect did not reverse aloin-induced PI3Kα inhibition in the combination group. The final biological effect of the combination of aloin and rapamycin was still the upregulation of autophagy and inhibition of osteosarcoma cells. In contrast, the synergy of aloin and rapamycin resulted in a stronger apoptosis effect and autophagy flux induction. Therefore, we believe that aloin has a higher regulatory priority on PI3K. The upregulation of AKT and mTOR phosphorylation might be a feedback effect during the regulation process or crosstalk that needs further interaction study.

The results of animal model experiments showed that aloin significantly inhibited the growth rate of osteosarcoma after 4 weeks of administration, and its therapeutic effect was slightly inferior to that of the cisplatin chemotherapy group. The autophagy marker LC3B was significantly upregulated, indicating that aloin induced autophagic flux in osteosarcoma cells in vivo. In the aloin group, the expression level of PI3Kα was significantly downregulated, while phosphorylated mTOR and phosphorylated AKT levels were significantly increased. This indicated that aloin induced autophagy and apoptosis of osteosarcoma through the PI3K/AKT/mTOR pathway in vivo. HE staining microscopic examination of vital organs also found no significant pathological changes in the aloin group.

## Conclusion

Aloin dose-dependently inhibited osteosarcoma proliferation and increased the autophagic flux. Network pharmacology and bioinformatics research inferred that aloin may inhibit osteosarcoma through the PI3K/AKT/mTOR pathway. Subsequent studies verified that aloin mediated the autophagic flux increase and osteosarcoma apoptosis through the PI3K/AKT/mTOR axis. As an inhibitor of autophagy, chloroquine reversed the autophagic flux increase with aloin. Meanwhile, starvation and rapamycin cooperated with the autophagy-inducing function of aloin. An osteosarcoma xenograft model showed that aloin inhibited osteosarcoma and mediated the PI3K/AKT/mTOR pathway in vivo, with no adverse reactions in major organs. We may infer that aloin may have the potential as an adjuvant drug for chemotherapy in osteosarcoma treatment.

## Supplementary Information

**Additional file 1.** Data declaration.

**Additional file 2**.

**Additional file 3**.

## Data Availability

The datasets generated and/or analysed during the current study are available in the Mendeley repository, https://data.mendeley.com/datasets/wn45tscc28/draft?a=b4172ac4-f662-49b6-9ec3-cc88bd13de23**(**Further inquiries can be directed to the corresponding authors).

## Abbreviations

AKT: Protein kinase B
ATG: Autophagy related gene
DMEM: Dulbecco’s modified Eagle’s medium
DMSO: Dimethylsulfoxide
EGFR: Epidermal Growth Factor Receptor
FACS: Fluorescence Activating Cell Sorter
FITC: Fluorescein Isothiocyanate
HE: Hematoxylin–eosin
HRP: Horseradish Peroxidase
IHC: Immunohistochemistry
IVIS: In vivo Bioluminescence imaging system
KEGG: Kyoto Encyclopedia of Genes and Genomes
MAPK: Mitogen-activated protein kinase
mTOR: Mammalian target of rapamycin
MTT: 3-(4,5)-Dimethylthiahiazo(-2)-3,5-diphenytetrazoliumromide
PARP: Poly ADP-ribose polymerase
PBS: Phosphate buffered saline
PI: Propidium Iodide
PI3K: Phosphatidylinositol 3 kinase
PPI: Protein–protein interaction
SD: Standard Deviation
SQSTM: Sequestosome
UCSF: University of California, San Francisco
