# Supplementary material for: Aloin induced apoptosis by enhancing autophagic flux through the PI3K/AKT axis in osteosarcoma
Source: Chin Med. 2021 Nov 24;16:123. doi: 10.1186/s13020-021-00520-4 (PMC8611986; doi:10.1186/s13020-021-00520-4)
Supplement: Supplementary file 1 — Additional file 1. Data declaration. [file 13020_2021_520_MOESM1_ESM.pdf]

Additional file 1: Data declaration.

The uncropped graph of western blotting, fluorescent microscope graph and network pharmacology parameters were uploaded to Mendeley Data website.

Please visit the below website to get the shared data: https://data.mendeley.com/datasets/wn45tscc28/draft?a=b4172ac4-f662-49b6-9ec3-cc88bd13de23

Cite information: He, Jiaming (2021), "Aloin research dataset ", Mendeley Data, V1, doi: 10.17632/wn45tscc28.1

If any difficult exists in visiting these data, please send E-mail to inform us. We will provide these graph in other methods.
