# Supplementary figures and images for: Aloin induced apoptosis by enhancing autophagic flux through the PI3K/AKT axis in osteosarcoma
Source: Chin Med. 2021 Nov 24;16:123. doi: 10.1186/s13020-021-00520-4 (PMC8611986; doi:10.1186/s13020-021-00520-4)

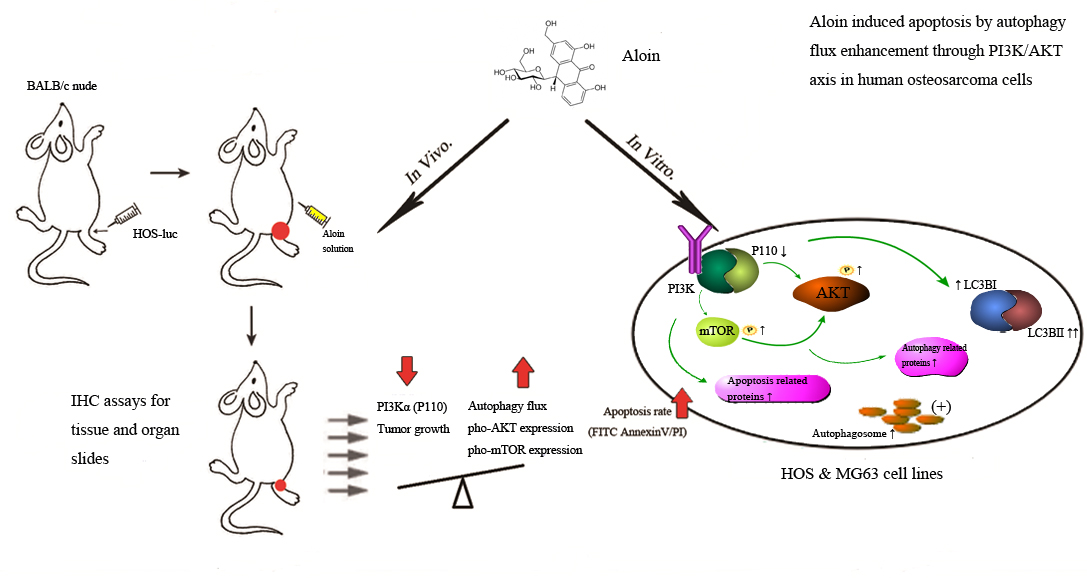

Supplement: Supplementary file 2 — Additional file 2. [file 13020_2021_520_MOESM2_ESM.jpg]
