# Supplementary material for: Aloin induced apoptosis by enhancing autophagic flux through the PI3K/AKT axis in osteosarcoma
Source: Chin Med. 2021 Nov 24;16:123. doi: 10.1186/s13020-021-00520-4 (PMC8611986; doi:10.1186/s13020-021-00520-4)
Supplement: Supplementary file 3 — Additional file 3. [file 13020_2021_520_MOESM3_ESM.docx]

### Highlights

- Natural anthraquinone glycoside from Aloe vera inhibit osteosarcoma *in vivo* and *in vitro*.
- Screening possible targets and pathways by network pharmacology and molecular docking
- Autophagy flux enhancement in human osteosarcoma cells
- PI3Kα downregulation of Aloin induced autophagy related effects and verified by turn-over experiments
- Xenograft osteosarcoma model for in vivo histological examination.
